# Generation of two genomic-integration-free DMD iPSC lines with mutations affecting all dystrophin isoforms and potentially amenable to exon-skipping

**DOI:** 10.1016/j.scr.2019.101688

**Published:** 2020-03

**Authors:** Giulia Ferrari, Francesco Muntoni, Francesco Saverio Tedesco

**Affiliations:** a Department of Cell and Developmental Biology, University College London, WC1E 6DE London, United Kingdom; b Dubowitz Neuromuscular Centre, Great Ormond Street Institute of Child Health, University College London, London WC1N 1EH, United Kingdom; c NIHR Great Ormond Street Hospital Biomedical Research Centre, London, United Kingdom

## Abstract

Duchenne muscular dystrophy (DMD) is the most common paediatric muscular dystrophy and is caused by mutations in the *DYSTROPHIN* gene. We generated two induced pluripotent stem cell (iPSC) lines from DMD patients with nonsense mutations in exons 68 (UCLi011-A) or 70 (UCLi012-A) by transfecting reprogramming mRNAs. Both mutations affect expression of all dystrophin isoforms. iPSCs expressed pluripotency-associated markers, differentiated into cells of the three germ layers in vitro and had normal karyotypes. The selected mutations are potentially amenable to read-through therapies, exon-skipping and gene-editing. These new iPSCs are also relevant to study DYSTROPHIN role in tissues other than skeletal muscle.

**Resource Table**

**Table:** 

Unique stem cell lines identifier	UCLi011-A
	UCLi012-A
Alternative names of stem cell lines	DMD iPSCs ex.68 (UCLi011-A)
	DMD iPSCs ex.70 (UCLi012-A)
Institution	University College London (UCL), London, UK
Contact information of distributor	Dr Francesco Saverio Tedesco (f.s.tedesco@ucl.ac.uk)
Type of cell lines	iPSCs
Origin	Human
Cell Source	Fibroblasts
Clonality	Mixed
Method of reprogramming	Transgene free (mRNA transfection)
Multiline rationale	Same disease non-isogenic cell lines
Gene modification	Yes
Type of modification	Spontaneous mutation
Associated disease	Duchenne muscular dystrophy
Gene/locus	DMD Cells c.9851G>*A* (p.Trp3284X) in exon 68
	DMD Cells c.10141C>*T* (p.Arg3381X) in exon 70
Method of modification	N/A
Name of transgene or resistance	N/A
Inducible/constitutive system	N/A
Date archived/stock date	15/04/2019
Cell line repository/bank	Human Pluripotent Stem Cell Registry (hpscreg.eu):
	• https://hpscreg.eu/cell-line/UCLi012-A (Biosample SAMEA5574041)
	• https://hpscreg.eu/cell-line/UCLi011-A (Biosample SAMEA5574032)
Ethical approval	Fibroblasts were obtained from the MRC Neuromuscular center Biobank (UCL, London, UK; Research Ethics Committee reference no. 06/Q0406/33). Human cell work was conducted under the approval of the National Health Service (NHS) Health Research Authority Research Ethics Committee reference no. 13/LO/1826; Integrated Research Application System (IRAS) project no. 141,100.

## 1 Resource utility

The new genomic-integration-free DMD iPSC lines UCLi011-A and UCLi012-A carry nonsense mutations beyond exon 63 of the dystrophin gene (Table 1). Although uncommon, mutations located between exon 63 and exon 79 cause loss of all the dystrophin isoforms including Dp71, the most abundant isoform in brain, and which deficiency is highly associated with cognitive impairment. These mutations are potentially amenable to therapeutic approaches based upon exon-skipping and read-through strategies (reviewed in Scoto et al., 2018) and are relevant to study dystrophin role both in muscle and extra-muscular tissues. Overall, UCLi011-A and UCLi012-A iPSCs will be useful to study the impact of dystrophin deficiency in multiple tissues and to screen possible therapies, particularly using recently-established platforms of complex muscle disease modelling in vitro (Maffioletti et al., 2018).

**Table 1:** Summary of lines.

iPSC line names	Abbreviation in figures	Gender	Age	Ethnicity	Genotype of locus	Disease
UCLi011-A	DMD iPSCs ex.68	Male	8	N/A	DMD Cells c.9851G>*A* (p.Trp3284X) in exon 68	DMD
UCLi012-A	DMD iPSCs ex.70	Male	3	N/A	DMD Cells c.10141C>*T* (p.Arg3381X) in exon 70	DMD

## 2 Resource details

DMD is an inherited muscle-wasting disorder of childhood caused by mutations in the dystrophin gene (Mercuri and Muntoni 2013). Dystrophin is the largest gene in nature and has a very complex transcriptional regulation, with several tissue specific isoforms associated with their own promoter and unique first exon (Muntoni et al., 2003). The deficiency of Dp71, the shortest isoform, although ubiquitously expressed, has been linked to severe cognitive deficit, thus raising interest in the function it plays in the central nervous system. The promoter and unique first exons of this isoform is located in intron 62 of the dystrophin gene, so any mutations located towards the 3′ of exon 63 will affect Dp71 in addition to all the remaining isoforms.

We generated two iPSC lines starting from skin fibroblasts from two DMD patients aged 8 (iPSC UCLi011-A) and 3 (iPSC UCLi012-A) with nonsense mutations in exon 68 and 70, respectively (kindly provided by the MRC Neuromuscular Biobank, London). Fibroblasts were reprogrammed into iPSCs via serial transfections with a mix of mRNAs encoding the reprogramming factors OCT4, SOX2, KLF4, CMYC, NANOG and LIN28, as well as reprogramming-enhancing microRNAs (microRNA-enhanced mRNA reprogramming protocol; Stemgent, cat. No. 00–0071 and 00–0073).

**Fig. 1:**
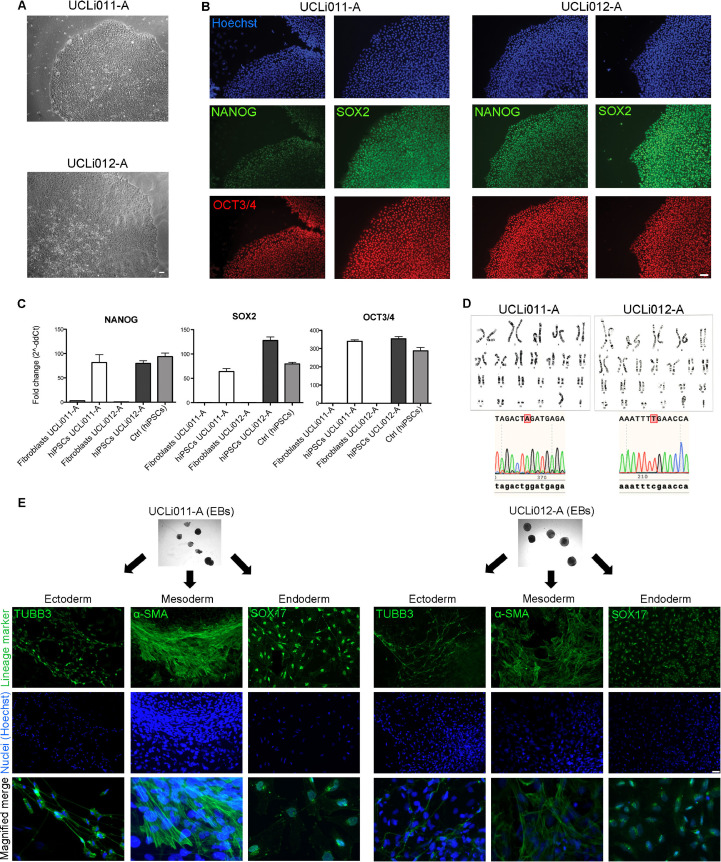
(A) Phase contrast images showing colonies of both DMD iPSC lines. Scale bar 100 μm. (B) Immunofluorescence staining showing pluripotency-associated markers (NANOG, SOX2 and OCT3/4) in UCLi011-A and UCLi012-A iPSCs. Hoechst: nuclei. Scale bar: 75 μm. (C) Quantitative real-time PCR analysis showing expression of mRNAs of pluripotency-associated factors (*NANOG, SOX2* and *OCT3/4*) in UCLi011-A and UCLi012-A iPSCs and their absence in parental fibroblasts. (D) Upper images: normal karyotype of UCLi011-A and UCLi012-A iPSCs (46,XY). Lower images: electropherograms confirming presence of pathogenic mutations in exon 68 (c.9851G>*A* (p.Trp3284X) and 70 c.10141C>*T* (p.Arg3381X) of the dystrophin gene. (E) Embryoid body formation assay. Upper phase contract images show morphology of UCLi011-A and UCLi012-A embryoid bodies. Lower panels show immunofluorescence staining of the same embryoid bodies with lineage-specific markers: α-smooth muscle actin (mesoderm), βIII-tubulin (ectoderm) and SOX17 (endoderm). Hoechst: nuclei. Scale bar: 75 μm. Bottom images: merged pictures showing magnified areas of each lineage-specific staining.

The resulting iPSC lines showed the expected morphology of human pluripotent colonies (Fig. 1A) and expressed the pluripotency-associated markers OCT4, NANOG and SOX2 at mRNA and protein levels (Fig. 1B,C). Both iPSCs UCLi011-A and UCLi012-A presented a normal karyotype (46,XY) with a correct ploidy and no major chromosomal abnormalities (Fig. 1D; UCLi011-A tested at passage 8; UCLi012-A tested at passage 6). Sanger sequencing confirmed that after reprogramming the DMD iPSCs still harbour the disease-causing mutations located in exon 68 (c.9851G>*A* (p.Trp3284X) and 70 c.10141C>*T* (p.Arg3381X) of the dystrophin gene (Fig. 1D). Functional pluripotency was demonstrated by differentiation into cell types of the three germ layers in embryoid body formation assays (Fig. 1E).

Additionally, cell identity was confirmed by STR analysis, which demonstrated a 100% match in the analysed alleles of parental fibroblasts and derived iPSCs (available with the authors). Finally, both iPSC lines were negative for Mycoplasma contamination (characterization and validation summarized in Table 2).

**Table 2:** Characterization and validation.

Classification	Test	Result	Data
Morphology	Photography (phase contrast microscopy)	Adherent colonies with epithelial morphology and high nuclear-cytoplasmic ratio.	Fig. 1 panel A
Phenotype	Qualitative analysis (Immunofluorescence)	Positive staining for pluripotency-associated markers: OCT4, NANOG, SOX2	Fig. 1 panel B
	Quantitative analysis (RT-qPCR)	Positive expression of *OCT4, NANOG AND SOX2* transcripts	Fig. 1 panel C
Genotype	Karyotype (G-banding) and resolution	46XY, Resolution 6–10 MB	Fig. 1 panel F
Identity	STR analysis	16 loci tested; 100%match	Summary table available with authors
Mutation analysis (IF APPLICABLE)	Sequencing	X-linked mutations: DMD c.9851G>*A* (p.Trp3284X) in exon 68; DMD c.10141C>*T* (p.Arg3381X) in exon 70	Fig. 1 panel D
Microbiology and virology	Mycoplasma	Mycoplasma testing by luminescence: Negative	Materials and methods
Differentiation potential	Embryoid body formation	Embryoid bodies spontaneous differentiation: α-smooth muscle actin (mesoderm), βIII-tubulin (ectoderm) and SOX17 (endoderm)	Fig. 1 panel E

## 3 Materials and methods

### 3.1 Cell culture

DMD fibroblasts were cultured in Dulbecco's modified Eagle's medium (DMEM) supplemented with 10% foetal bovine serum (FBS; Sigma) and 1% penicillin-streptomycin antibiotics (PS) (Sigma). 5 × 10^4^ fibroblasts were plated onto 6-cm dishes coated with Matrigel™. After 24 h, media was changed to NuFF-conditioned Pluriton media supplemented with B18R (eBioscience) and Pluriton supplement (Stemgent). Subsequently fibroblasts were transfected with a mix of mRNA reprogramming factors (OCT4, SOX2, KLF4, cMYC, NANOG and LIN28; Stemgent, cat.no 00–0071) (Warren et al. 2010)**,** following manufacturer instructions. Transfections were performed daily for 11 days with Stemgent Stemfect RNA Transfection Kit (Stemgent 00–0069) in NuFF-conditioned Pluriton media; cells were not split/passaged until appearance of colonies. On days 1 and 5, MicroRNAs (Stemgent, cat.no. 00–0073) were added to the mRNA cocktail to enhance reprogramming efficiency, following manufacturer's instructions.

From day 19, the first colonies were picked and plated onto 6-well dishes. iPSCs UCLi011-A were plated directly on Vitronectin XF™(Stemcell Technologies), and maintained in feeder-free, chemically defined TeSR™-E8™ medium (Stemcell Technologies, cat.no. 05,940) at 37 °C with 5% CO2 and 3% O2. Approximately every 6 days, iPSCs were passaged via either manual picking or gentle cell dissociation reagent at a 1:8 ratio (Stemcell Technologies, cat. no.07174), following manufacturer's instructions. iPSCs UCLi012-A were first expanded on feeder cells (mouse embryonic fibroblasts) to increase their attachment and viability. After two passages on feeders, iPSCs UCLi012-A were stabilised in feeder-free conditions (~2/3 passages) as described for iPSCs UCLi011-A. Mycoplasma contamination was ruled out by MycoAlert™ kit, following manufacturer's instructions (Lonza); a ratio <0.9 is considered negative: UCLi011-*A* = 0.48; UCLi012-*A* = 0.71.

### 3.2 Immunofluorescence

Cells were washed with PBS, fixed with 4% (w/v) PFA for 5 min, followed by a further PBS wash. Fixed cells were permeabilized for 1 hour with permeabilization solution (1% bovine serum albumin (BSA) + 0.2% Triton in PBS) at room temperature. Cells were then blocked for 30 min with 10% donkey or goat serum diluted in permeabilizing solution at room temperature. Cells were then incubated overnight at 4 °C with the primary antibodies (Table 3) diluted in permeabilization solution. Unbound primary antibody was removed with three washes of 0.2% Triton in PBS. Cells were then incubated for 1 hour with secondary antibodies and Hoechst 33342 diluted in 0.2% Triton in PBS. Unbound secondary antibody was washed away with two washes of 0.2% Triton in PBS, followed by one rinse with PBS. Cells were imaged with an inverted fluorescence microscope (Leica DM16000B).

### 3.3 qPCR analysis

RNA was isolated from cell pellets using RNeasy Mini kit (Qiagen; 74,104) according to manufacturer's instructions. A DNase step was included to eliminate genomic contamination. RNA quality and yield was assessed using a Nanodrop. Retro-transcription to cDNA was conducted with the ImProm-II™ Reverse Transcription System kit (Promega; A3800) following manufacturer's instructions. qPCRs were performed with SYBR-Green Real Time Master Mix (Promega; A600A) according to manufacturer instructions using the BioRad CFX96 machine. A house keeping gene (GAPDH) reaction was included on each plate for all samples. The ΔCT method has been used to analyse the experimental CT values. A commercially-available human iPSC line (Gibco; cat. no. A13777) has been included as a positive control and human myoblasts provided a negative control. Primer sequences are listed in Table 3.

**Table 3:** Reagents details.

Antibodies used for immunocytochemistry/flow-citometry			
	Antibody	Dilution	Company Cat # and RRID
Pluripotency Markers	Mouse anti-OCT4	1:100	Santa Cruz Biotechnology Cat# sc-5279, RRID:AB_628,051
Pluripotency Markers	Rabbit anti-SOX2	1:200	Abcam cat# ab97959 AB_2,341,193
Pluripotency Markers	Rabbit anti-NANOG	1:200	Abcam cat# ab80892 RRID: AB_2,150,114
Differentiation Markers	Rabbit anti- SOX17	1:100	Millipore cat#09–038 RRID: AB_1,587,525
Differentiation Markers	Mouse anti-Actin, α-smooth muscle	1:300	Sigma cat# A2547 RRID: AB_476,701
Differentiation Markers	Mouse anti class III beta-Tubulin	1:100	Stemcell Technologies cat# 1409 RRID: AB_215,509
Secondary antibodies	Donkey Anti-Mouse IgG, Alexa Fluor 546	1:500	Thermo Fisher Scientific Cat# A10036, RRID:AB_2,534,012
Secondary antibodies	Donkey Anti-Rabbit IgG, Alexa Fluor 647	1:500	Molecular Probes Cat# A-31,573, RRID:AB_2,536,183
Secondary antibodies	Donkey Anti-Mouse IgG, Alexa Fluor 488	1:500	Molecular Probes Cat# A-21,202, RRID:AB_141,607

Primers			
	Target	Forward/Reverse primer (5′−3′)	
Targeted mutation analysis/sequencing	DMD c.9851G>*A* (p.Trp3284X) in exon 68	CCAGCCTAGCTTTGCAACCAT / CCCGTGAAGACACGCACT	
Targeted mutation analysis/sequencing	DMD c.10141C>*T* (p.Arg3381X) in exon 70	CCTGGTTTCAGAGCCCCATT / TGGCAACTGGACATCAGCTT	
House-Keeping Gene (qPCR)	GAPDH	TTCACCACCATGGAGAAGGC/ GGCATGGACTGTGGTCATGA	
Reprogramming factor (qPCR)	NANOG	CAATGGTGTGACGCAGGGAT/ CCAAGTCACTGGCAGGAGAAT	
Reprogramming factor (qPCR)	SOX2	AACCAGCGCATGGACAGTTA/ GACTTGACCACCGAACCCAT	
Reprogramming factor (qPCR)	OCT3/4	AGGTTTCTCACCTGTGTGGGTT/ CTTTGTGTTCCCAATTCCTTCC	

### 3.4 Embryoid body formation assay

iPSCs were dissociated into clumps using gentle cell dissociation reagent and embryoid bodies (EBs) were allowed to form and grow in suspension in TeSR™-E6 medium (Stemcell Technologies) in non-adhesive dishes. After 7 days EBs were transferred to standard 10 cm tissue cultures dishes to allow adhesion in DMEM (Sigma) with 20%(v/v) FBS (Life technologies), 1% l-glutamine (Sigma), 1% PS (Sigma) in 3% O_2_ and 5% CO_2_ to induce spontaneous differentiation. Media was changed every other day and plates were fixed in 4% PFA after 14–20 days of differentiation.

### 3.5 Sequencing, STR profiling and karyotype analysis

Genomic DNA was extracted from each cell line by DNeasy kit (Quiagen). 100 ng/μl of Gotaq® DNA polymerase (Promega) was used for amplification (35 cycles using a BioRad T100™ Thermal cycler). DMD specific primers upstream and downstream the point mutations were designed (Table 3) and purified PCR reactions sequenced via dideoxynucleoside Sanger sequencing by Source Biosciences (Cambridge). iPSCs UCLi011-A and UCLi012-A were authenticated by STR analysis performed by Source Biosciences (Nottingham) using Promega PowerPlex 16 HS assay (available with the authors). For each cell line karyotyped, a T25 flask of 80% confluent cells was sent to The Doctors Laboratory (TDL, London, UK) were G-band analysis was performed at a resolution of 6–10 MB (UCLi011-A: passage 8, 10 metaphase spreads analysed; UCLi012-A: passage 6, 20 metaphase spreads analysed).
